# Multiple introduced lineages and the single native lineage co-driving the four waves of the COVID-19 pandemic in West Africa

**DOI:** 10.3389/fpubh.2022.957277

**Published:** 2022-09-15

**Authors:** Liping Gao, Canjun Zheng, Qi Shi, Lili Wang, Alie Tia, Jone Ngobeh, Zhiguo Liu, Xiaoping Dong, Zhenjun Li

**Affiliations:** ^1^State Key Laboratory of Infectious Disease Prevention and Control, National Institute for Viral Disease Control and Prevention, Chinese Center for Disease Control and Prevention, Beijing, China; ^2^Sierra Leone-China Friendship Biological Safety Laboratory, Freetown, Sierra Leone; ^3^Chinese Center for Disease Control and Prevention, Beijing, China; ^4^State Key Laboratory of Infectious Disease Prevention and Control, National Institute for Communicable Disease Control and Prevention, Chinese Center for Disease Control and Prevention, Beijing, China

**Keywords:** COVID-19, epidemiological, cases, deaths, West Africa, four waves

## Abstract

Coronavirus disease 2019 (COVID-19) has become a vast burden on public health and socioeconomics in West Africa, but the epidemic situation is unclear. Therefore, we conducted a retrospective analysis of the positive rate, death rate, and diversity of SARS-CoV-2. As of March 31, 2022, a total of 894,813 cases of COVID-19 have been recorded, with 12,028 deaths, both of which were distributed in all 16 countries. There were four waves of COVID-19 during this period. Most cases were recorded in the second wave, accounting for 34.50% of total cases. These data suggest that although West Africa seems to have experienced a low and relatively slow spread of COVID-19, the epidemic was ongoing, evolving with each COVID-19 global pandemic wave. Most cases and most deaths were both recorded in Nigeria. In contrast, the fewest cases and fewest deaths were reported, respectively, in Liberia and Sierra Leone. However, high death rates were found in countries with low incidence rates. These data suggest that the pandemic in West Africa has so far been heterogeneous, which is closely related to the infrastructure of public health and socioeconomic development (e.g., extreme poverty, GDP per capita, and human development index). At least eight SARS-CoV-2 variants were found, namely, Delta, Omicron, Eta, Alpha, Beta, Kappa, Iota, and Gamma, which showed high diversity, implicating that multiple-lineages from different origins were introduced. Moreover, the Eta variant was initially identified in Nigeria and distributed widely. These data reveal that the COVID-19 pandemic in the continent was co-driven by both multiple introduced lineages and a single native lineage. We suggest enhancing the quarantine measures upon entry at the borders and implementing a genome surveillance strategy to better understand the transmission dynamics of the COVID-19 pandemic in West Africa.

## Background

Coronavirus disease 2019 (COVID-19) is caused by Severe Acute Respiratory Syndrome Coronavirus 2 (SARS-CoV-2), which has caused a large global outbreak and is a major public health concern (1). Due to alarming levels of spread and severity, on March 11, 2020, the World Health Organization (WHO) declared the outbreak as a global pandemic. As of March 31, 2022, COVID-19 has affected 225 countries and territories, and 487,232,852 cases of COVID-19 have been recorded, with 6,163,223 cases of death and 422,461,956 recoveries worldwide (https://www.worldometers.info/). Moreover, 11,756,052 cases of COVID-19 have been recorded, with 252,811 cases of death and 10,975,288 recoveries, in Africa, of which the confirmed cases and deaths accounted for 2.4 and 4.1%, respectively. Most of the initial COVID-19 cases in African countries were imported cases from Europe (2). Around January 28, 2020, Nigeria (NGA) announced sub-Saharan Africa's first confirmed case of COVID-19 (3), and the remaining 15 West African countries detected their first cases all in March 2020. West Africa is the most underdeveloped area in Africa. The region includes nine of the 25 poorest countries in the world (4). These countries have poorly resourced healthcare systems; so, a rapid increase in the number of cases could quickly overwhelm the already vulnerable healthcare systems (5, 6). Moreover, HIV, tuberculosis, and malaria are the three most important infectious diseases in West Africa. With the fragile economic conditions and healthcare systems in the region, intervention measures such as lockdown and quarantine can be more catastrophic than the disease itself (7). The COVID-19 pandemic resulted in disruptions of routine healthcare services and diversion of the already limited available resources in West Africa (8). Extreme poverty is rising in West Africa due to the COVID-19 pandemic and preventive measures (such as border closures and movement restrictions) deteriorating the economic situation, which adversely affected the income, access to healthcare services, and food security and drove prices up (9). Currently, more than 25 million people in West Africa are unable to meet their basic food needs, an increase of 34% compared to 2020 (10). Therefore, a comprehensive analysis of the pandemic spread patterns and the diversity of SARS-CoV-2 variants is essential to achieve a better balance, maintain social order, and keep the pandemic under control in West Africa.

A systematic interpretation of COVID-19 data will aid us to better understand the spread patterns and scale of the pandemic and will contribute to the formulation of targeted measures to mitigate the negative effects on health and stabilize the socioeconomic system (11, 12). The purpose of this study was to describe the epidemiological profile of COVID-19 and variant diversity in West Africa in order to generate evidence to further enhance planning and response strategies to further guide epidemiological interventions and the allocation of scarce resources based on the disease patterns.

## Methods

### Ethics statement

This study was supported by the China–Sierra Leone Biosafety Laboratory Technical Cooperation Project (Phase III) and was approved by the Commission of Ethics and Science Censor of the Sierra Leone Ministry of Health and Sanitation. Our survey adhered to the medical ethics of domestic laws and regulations.

### Data source, data processing, and wave definition

In our study, epidemiological data and information with respect to related social factors (e.g., reproduction rate (Rt) and stringency index) of COVID-19 in West Africa were extracted from a public COVID-19 surveillance dataset website, “Our World in Data” (https://ourworldindata.org/covid-cases), from March 2020 to March 2022. Data on SARS-CoV-2 variants from each country in West Africa were also obtained from a public database (https://www.gisaid.org/). The acquired data were then cleaned, processed, and analyzed using Microsoft Excel 2016 (Redmond, Washington, USA) and cross-checked by two trained qualified healthcare workers. The death rate was calculated using the following formula: death rate = deaths/confirmed cases × 100%. The month-average values of Rt and stringency index were calculated and then used for epidemic trend analysis. Subsequently, a systematic variables correlation analysis (Pearson correlation coefficient) was conducted by ChiPlot (https://www.chiplot.online/#Line-plot) to evaluate the association between sociodemographic factors (e.g., Rt, extreme poverty, Gdp per capita, and human development index, and age) and cases (confirmed cases and deaths). A *p*-value < 0.05 was considered statistically significant.

We used the study variable “wave,” which refers to a rising number of COVID-19 cases that has a specific peak and then declines. The waves were classified in this study as follows: the first wave, from March 2020 to September 2020; the second wave, from October 2020 to May 2021; the third wave, from June 2021 to October 2021; and the fourth wave, from November 2021 to March 2022.

## Results

### Epidemiological profile of COVID-19 in West Africa

**Figure 1:**
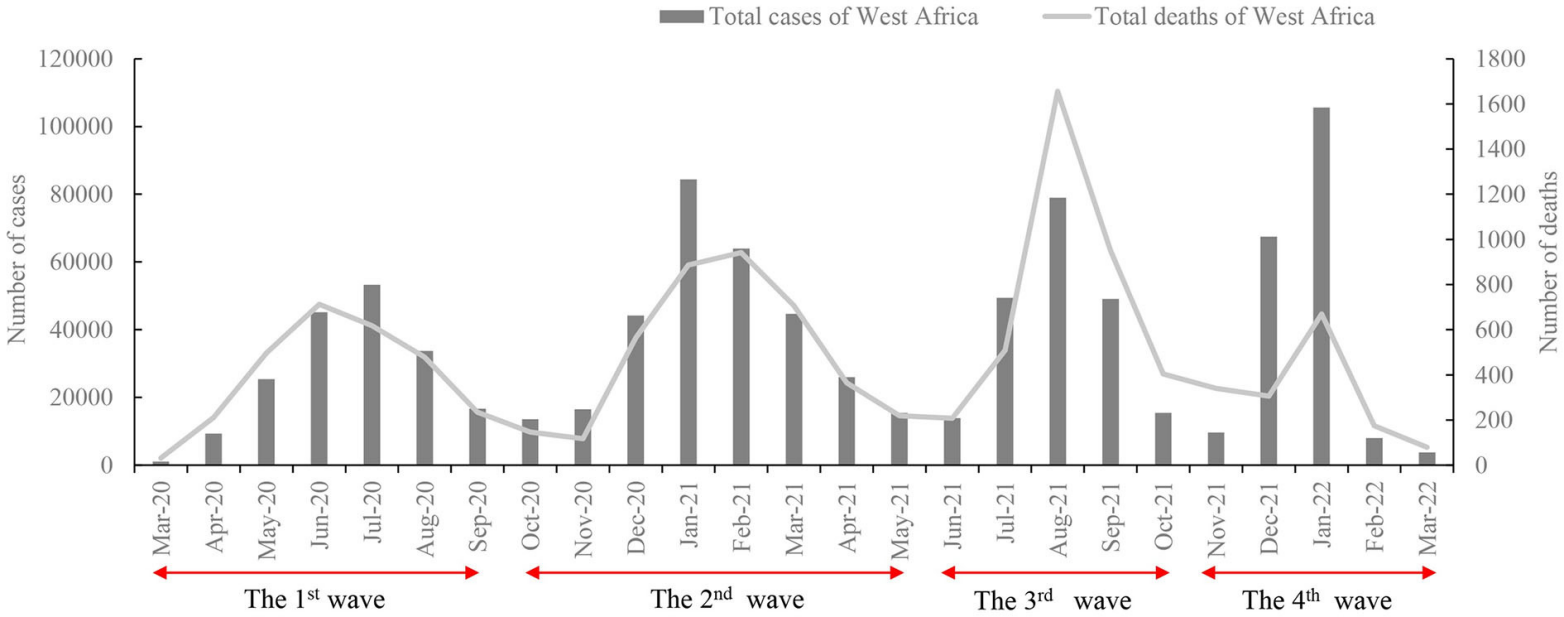
Evolution of cases and deaths in West Africa from March 2020 to March 2022.

As of March 31, 2022, a total of 894,813 cases of COVID-19 were recorded, with 12,028 deaths in all 16 countries in West Africa (Figure 1). There were four large waves of COVID-19 in West Africa during this period (Figure 1). The first wave was from March to September 2020; the second wave was from October 2020 to May 2021; the third wave was from June to October 2021; and the last wave was from November 2021 to March 2022. The numbers of confirmed cases in these four waves were 184,658, 308,698, 206,924, and 194,533, respectively. The second wave was the largest, accounting for 34.50% (308,698/894,813). Based on the month, the largest numbers of cases (*n* = 105,561) were reported in January 2022, and the lowest numbers were recorded in March 2020 (*n* = 1,061), with an average of 35,793 cases per month (Figure 1). The numbers of death cases in the four waves were 2,783, 3,947, 3,728, and 1,570, respectively, accounting for 32.82% of all deaths, with the largest number of death cases in the second wave and the lowest number in the fourth wave. Moreover, the largest number of death cases (*n* = 1,656) was noted in August 2021, while the lowest number was recorded in March 2020 (*n* = 31) (Figure 1).

### Geographic distribution of cases and deaths in West Africa

**Figure 2:**
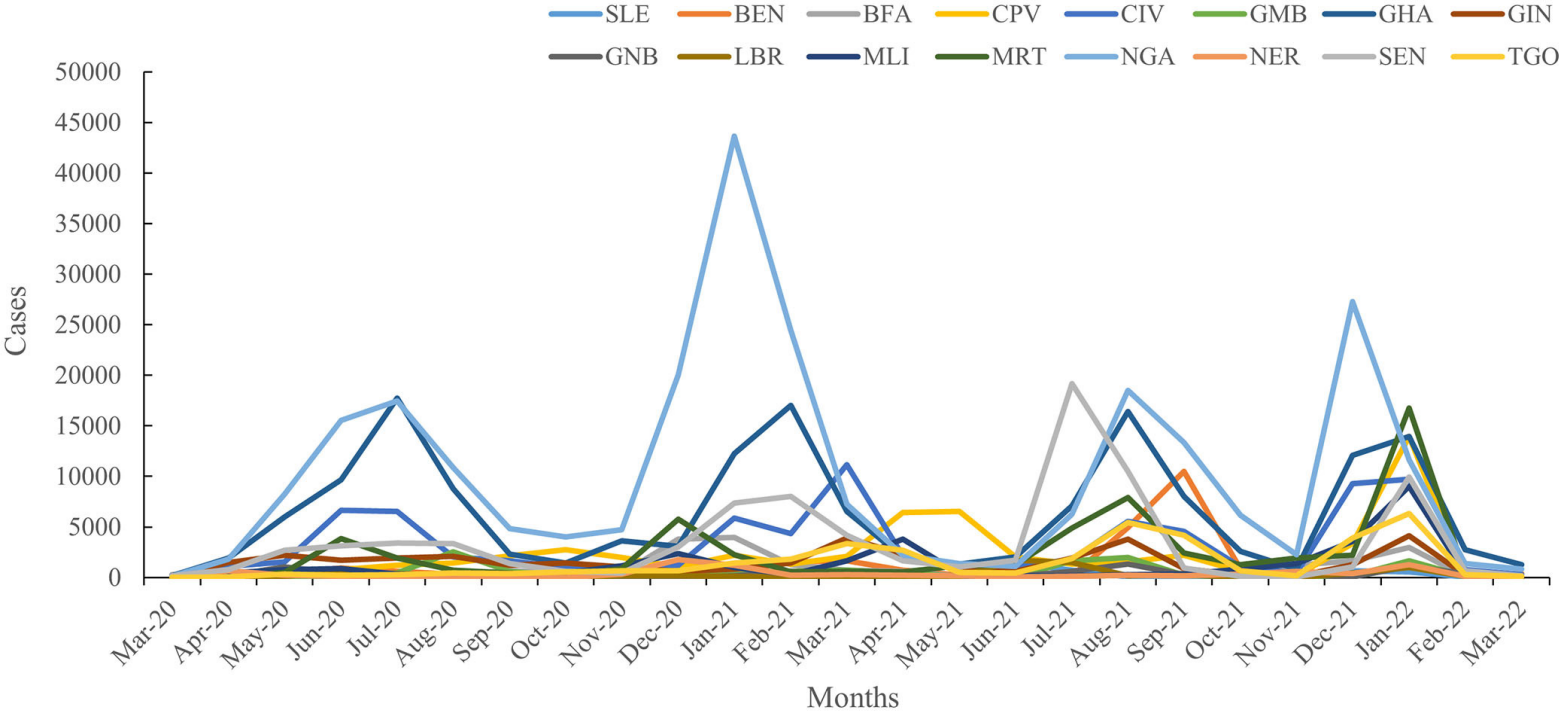
Epidemiological trends in the 16 countries in West Africa from March 2020 to March 2022.

**Figure 3:**
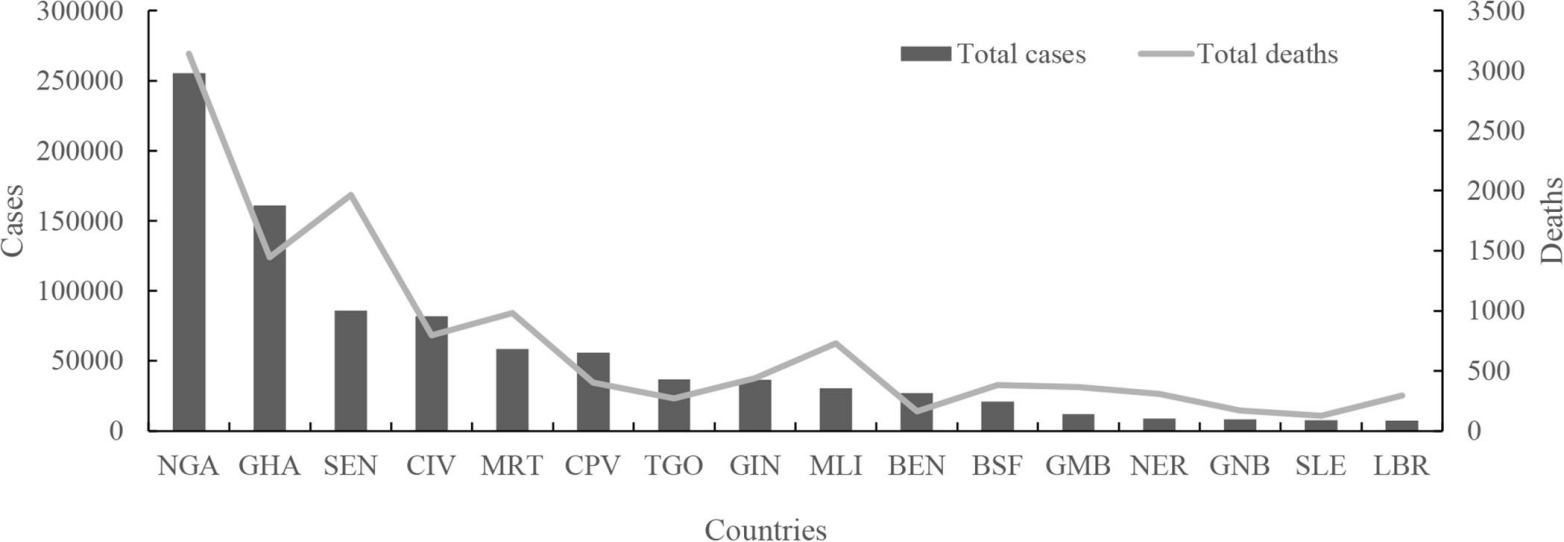
Distribution profile of the cases and deaths in West Africa.

**Figure 4:**
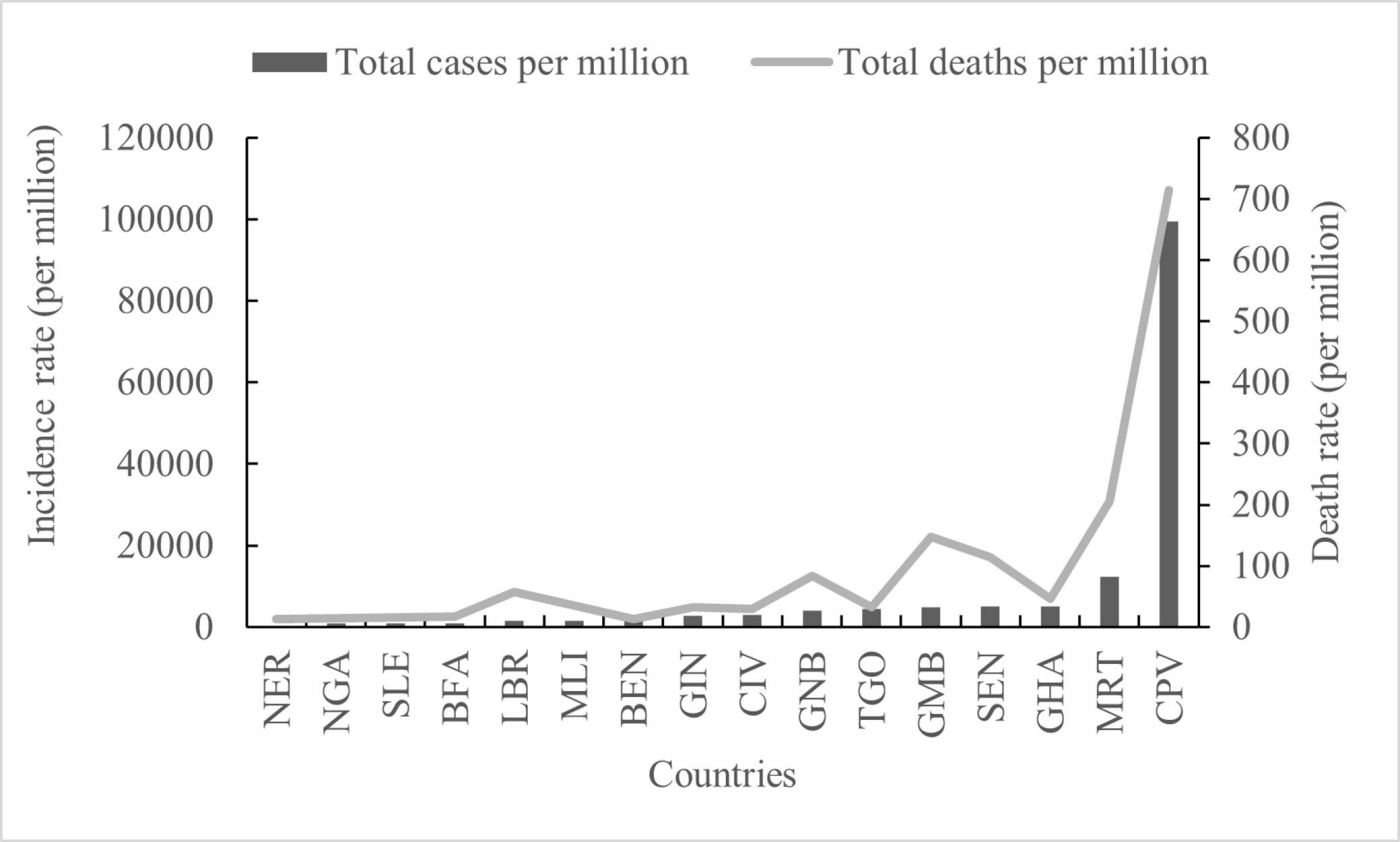
Comparative analysis of the total of the cases and deaths per million in 16 countries in West Africa.

Both confirmed cases and fatal cases were reported in all 16 countries in West Africa, and almost all countries experienced a four-wave COVID-19 epidemic (Figure 2). The numbers of confirmed COVID-19 cases from low to high were 7,526 in Liberia (LBR), 7,674 in Sierra Leone (SLE), 8,149 in Guinea-Bissau (GNB), 8,805 in Niger (NER), 12,088 in Gambia (GMB), 20,853 in Burkina Faso (BFA), 27,161 in Benin (BEN), 30,484 in Mali (MLI), 36,459 in Guinea (GIN), 36,939 in Togo (TGO), 55,952 in Cabo Verde (CPV), 58,668 in Mauritania (MRT), 81,775 in Côte d'Ivoire (CIV), 85,895 in Senegal (SEN), 160,971 in Ghana (GHA), and 255,414 in NGA (Figure 3) (Supplementary Table S1). Accordingly, the numbers of deaths from low to high were 125 in SLE, 163 in BEN, 170 in GNB, 272 in TGO, 294 in LBR, 308 in NER, 365 in GMB, 382 in BFA, 402 in CPV, 440 in GIN, 727 in MLI, 796 in CIV, 982 in MRT, 1,445 in GHA, 2,104 in SEN, and 3,143 in NGA (Figure 3) (Supplementary Table S1). The largest numbers of positive and fatal cases were recorded in NGA, whereas the lowest numbers were reported in LBR and SLE, respectively (Figure 3). Generally, higher crude fatal rates (CRFs) were identified in the countries with relatively few reported positive cases, with the lowest in BEN (13.091) and the highest in CPV (713.649) (Figure 4).

Based on the number of cases per million inhabitants, both the highest incidence and the highest death rate were recorded in CPV (99,494.395 and 713.649), and the lowest were recorded in NER (349.412 and 12.256) (Figure 4). The incidence rates in other regions were ordered from high to low as follows: MRT (12,284.115), GHA (5,064.772), SEN (4,989.269), GMB (4,810.335), TOG (4,346.892), GNB (4,002.997), CIV (3,016.12), GIN (2,698.849), BEN (2,134.361), MLI (1,458.592), LBR (1,426.97), BFA (965.293), and NGA (1208.199), BFA (965.293) and SLE (941.737) (Figure 4). However, there were obvious fluctuations in different countries in the total number of deaths per million inhabitants; high incidence and low death rates were observed in GHA and TGO, but low incidence and high death rates were observed in LBR, GNB, and GMB (Figure 4).

### The correlation between epidemics indexes (cases and deaths) and 17 sociodemographic factors

**Figure 5:**
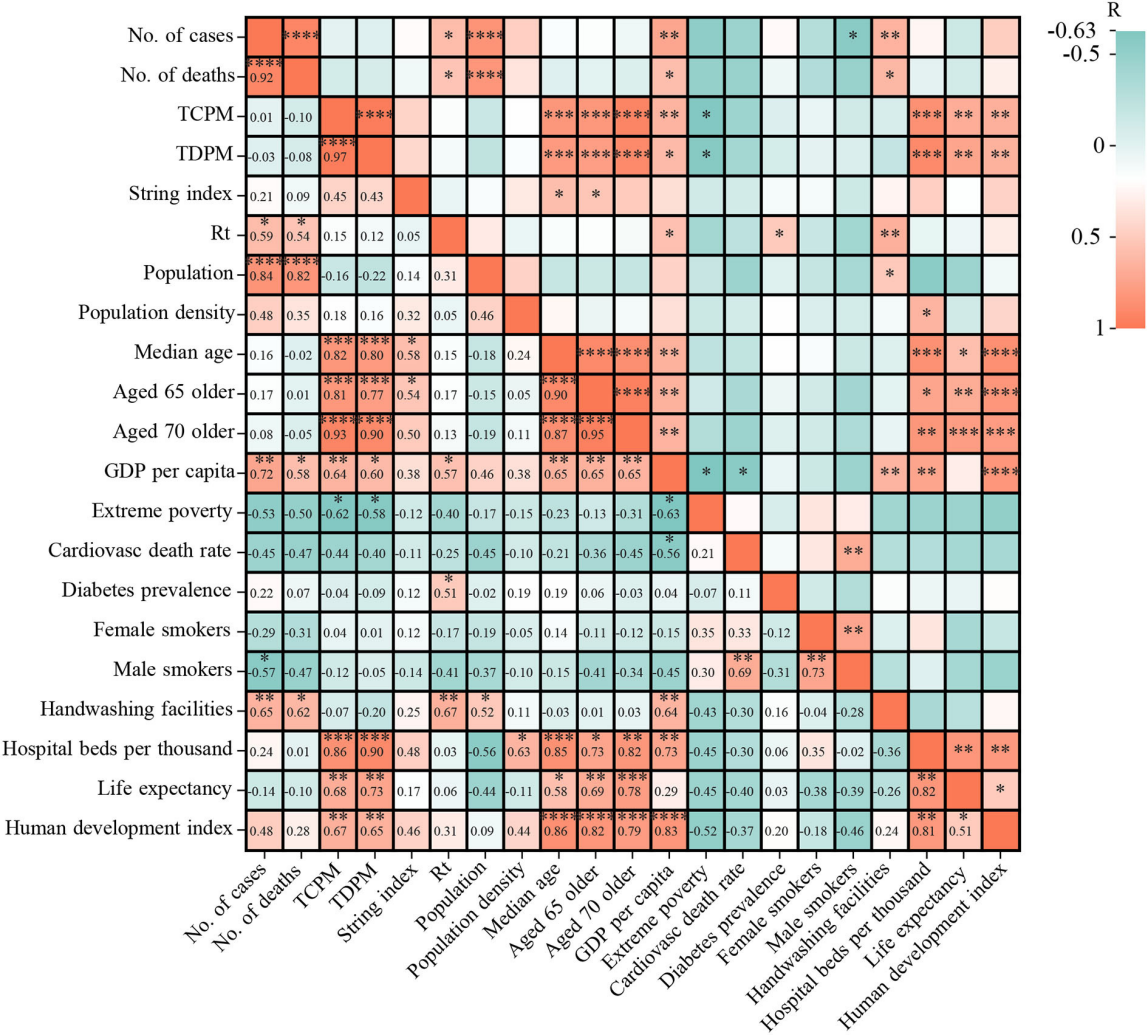
The correlation between epidemics indexes (cases and deaths) and 17 sociodemographic factors. Note: TCPM, total of cases per million; TDPM, total of deaths per million.

**Figure 6:**
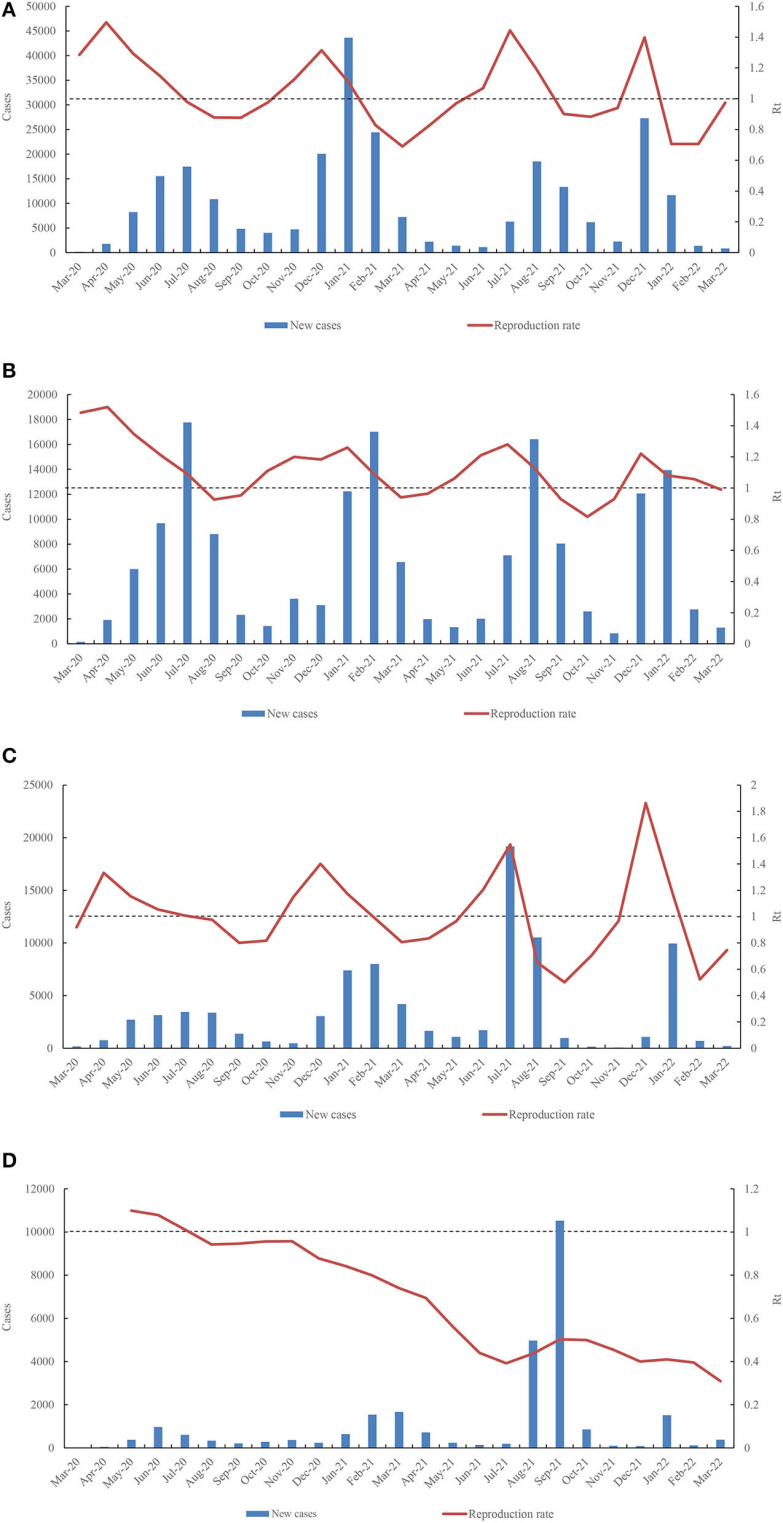
Dynamic fluctuation of Rt value in four countries **(A)** (NGA), **(B)** (GHA), **(C)** (SEN), and **(D)** (BEN) among the four waves COVID-19 pandemic.

Both of number of cases and number of deaths have significant correlation with population (CC ≥ 0.82, *p* < 0.005), handwashing facilities (correlation coefficient (CC) ≥ 0.62, *p* < 0.05), GDP per capita (CC ≥ 0.58, *p* < 0.05), and Rt (CC ≥ 0.54, *p* < 0.05) (Figure 5). In the initial phase of the COVID-19 pandemic, Rt was higher than 1.0 in 12 West African countries and lower than 1.0 in the remaining four countries, such as CPV (0.77), TGO (0.8), LBR (0.88), and SEN (0.93) (Supplementary Table S2). By March 31, 2022, only four countries showed relatively high Rt values (>1.0), i.e., NGA (Rt = 1.11), SEN (Rt = 1.06), TGO (Rt = 1.09), and MRT (Rt = 1.02) (Supplementary Table S2). The highest Rt values were frequently observed before every wave of COVID-19 in 15 countries, except for BEN. The Rt values in the early stages of the four waves in NGA were 1.496 (April 2020), 1.3145 (December 2020), 1.443 (July 2021), and 1.397 (December 2021) (Figure 6A). In GHA, the Rt values in the early stages of the four waves were 1.52 in April 2020, 1.26 in January 2021, 1.28 in July 2021, and 1.22 in December 2021 (Figure 6B), while those in SEN were 1.332 in April 2020, 1.401 in December 2020, 1.5484 in July 2021, and 1.863 in December 2021 (Figure 6C). However, the highest Rt value (1.09875) in BEN was recorded in May 2020, and it was low (< 1.0) by March 2022 (Figure 6D). Additionally, both of total of cases per million and the total of deaths per million had an obvious correlation with human development index (CC ≥ 0.65, *p* < 0.05), life expectancy (CC ≥ 0.68, *p* < 0.05), hospital beds per thousand (CC ≥ 0.86, *p* < 0.05), extreme poverty (CC ≥ 0.58, *p* < 0.05), GDP per capita (CC ≥ 0.60, *p* < 0.05), median age (CC ≥ 0.80, *p* < 0.05), aged 65 older (CC ≥ 0.77, *p* < 0.05), and aged 70 older (CC ≥ 0.90, *p* < 0.05) (Figure 5).

### Diversity and distribution profile of SARS-CoV-2 variants

**Figure 7:**
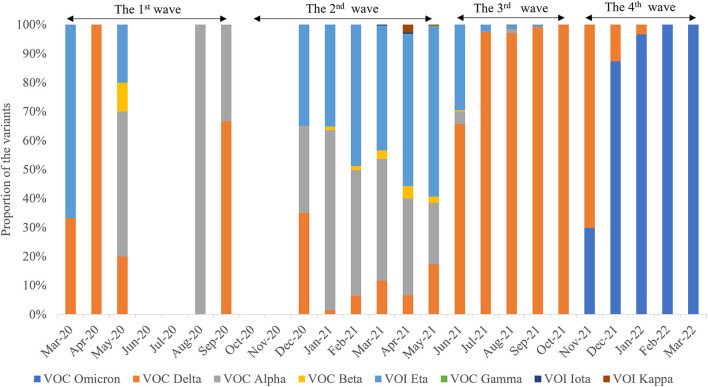
Spatiotemporal distribution characteristics of eight different SARS-CoV-2 variants during the four waves.

In total, 10,343 SARS-CoV-2 genomic sequences from West Africa have been submitted to the public database (https://www.gisaid.org/). As of March 31, 2022, at least eight SARS-CoV-2 variants were observed in West Africa, such as variant of concern (VOC) Delta (*n* = 5,636), VOC Omicron (*n* =1,933), variant of interest (VOI) Eta (*n* = 1,455), VOC Alpha (*n* = 1,233), VOC Beta (*n* = 68), VOI Kappa (*n* =13), VOI Iota (*n* = 4), and VOC Gamma (*n* = 1) (Figure 7). Of these, the VOC Delta variant was the most frequently identified lineage in West Africa, being detectable in 18 months (Figure 7).

**Figure 8:**
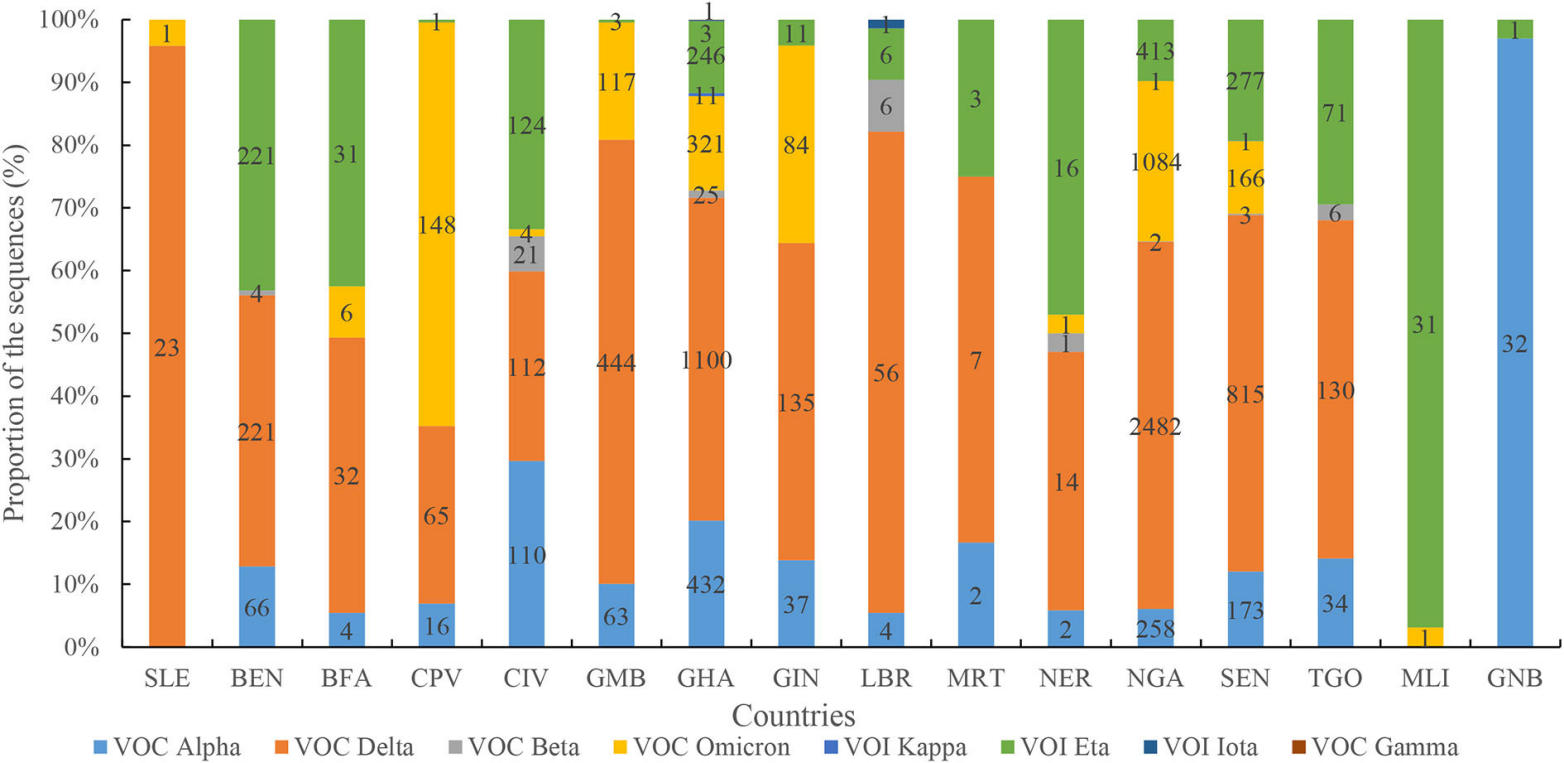
Diversity profile of SARS-CoV-2 variants in different countries. Note: figures in each column indicates number of the sequences in different SARS-CoV-2 variants.

The distributions of SARS-CoV-2 variants showed obvious spatiotemporal alterations in the four waves. In the first wave, the main VOCs were Alpha, Delta, Eta, and Beta. Seven variants were observed in the second wave, namely, Alpha, Delta, Eta, Beta, Kappa, Iota, and Gamma (Figure 8), in which Delta was the dominant lineage. Five variants were recorded in the third wave, namely, Delta, Alpha, Beta, Eta, and Kappa. Only Delta and Omicron were found in the fourth wave. Afterward, VOC Omicron was as the only lineage in West Africa (Figure 8). VOI Eta was identified in 15 countries (except for SLE), followed by VOC Delta in 14, VOC Alpha in 13, and VOC Omicron in 11. There were eight variants detected in GHA, six in NGA and SEN, five in LBR and NER, and four in BEN, BFA, CPV, GMB, GIN, and TGO.

## Discussion

A comprehensive retrospective analysis of the epidemiological evolution and variant diversity of COVID-19 in West Africa was conducted. Although the epidemic of the disease in West Africa has a low infection rate and seems to be spreading more slowly in West Africa than elsewhere, the COVID-19 pandemic has had severe health and socioeconomic impacts. More than 25 million people in West Africa are unable to meet their basic food needs, an increase of 34% compared to 2020. Because of this, people are selling their assets and livelihoods just to survive (13). However, the numbers of cases and deaths in Southern, Eastern, and Northern regions were higher than that in the Central and Western regions (14). Also, there were lower numbers of confirmed cases and deaths than in some developed countries, such as the United States, UK, Italy, and Brazil (https://covid19.who.int/). The following factors could contribute to this continent appearing to be more resistant to SARS-CoV-2 than other continents, these factors are a low testing rate, poor documentation of causes of death, a younger population, a lower population density, low income, less travel, a good vitamin D status as a result of exposure to sunlight, cross-immunity from other viruses (including coronaviruses), and lessons learned from other infectious diseases such as HIV and Ebola (15, 16). Indeed, most West Africans have basic knowledge of COVID-19 and show a positive attitude toward the disease (17). Importantly, the low incidences of SARS-CoV-2 infection and disease in sub-Saharan Africa appear to be correlated with the pre-pandemic serological cross-recognition of HCoVs, which are substantially more prevalent in sub-Saharan Africa than in the United States (18). Moreover, SARS-CoV-2 seroprevalence is high in both gold miners and administrative staff working in CIV. The burden of infection in West Africa has probably been underestimated (19). Therefore, some routine health measures are still necessary to balance between curbing the disease and sustaining the socio-economic order.

There were four waves of the COVID-19 pandemic in West Africa, which is similar to other African regions during this period. As of July 2021, some countries in Africa started experiencing spikes in the number of COVID-19 cases and related deaths, which is now referred to as the third wave (20). After the third wave peaked in July 2021, there has been a steady decline in new cases of COVID-19 across Africa, though a handful of countries are seeing resurgences (21). Africa's fourth pandemic wave was primarily driven by the Omicron variant. It is the most short-lived surge compared to the other three waves, with a 6-week increase (22).

In this study, the number of cases and deaths had a significant correlation with Rt, and the highest Rt value was observed before every wave in all 15 countries. These data suggest that there are several localized outbreaks in these countries. Similarly, a recent study showed that the Rt value at the start of the epidemic (mid-March 2020) was >3, quickly falling after the start of lockdown to a value <1 in late March 2020; fluctuation trends of the Rt value coincided with trends in the numbers of positive cases recorded (23).

Among the four waves of COVID-19 in West Africa during this period, the most severe wave was the second, accounting for 32.98% (295,108/894,813) of all cases. Similarly, although NGA took measures to manage a possible third wave, it was observed that the second wave was more severe than the first wave (24). The highest number of cases and the highest number of deaths were both recorded in NGA. In contrast, the lowest numbers of cases and deaths were reported in LBR and SLE, respectively. NGA is the most populous country in Africa and one of the largest metropolitan areas in the world. This might explain the high infectious rate in the region. Moreover, a recent report suggested that COVID-19 might not be spreading widely and that COVID-19 spread might be relatively curbed in SLE (25). However, a higher death rate was found in countries with fewer confirmed cases. These data suggest that the COVID-19 pandemic in various countries in West Africa is highly heterogeneous, which is closely related to the public healthcare infrastructure and the socioeconomic development in these countries. Our analysis showed that COVID-19 spread had an obvious correlation with multiple sociodemographics, such as human development index, life expectancy, hospital beds per thousand, extreme poverty, GDP per capita, median age, and so on. Similarly, a multivariate systematic correlation analysis showed that transmissibility of COVID-19 has a significant correlation with weather parameters, social development level, percent of the urban population, alcohol consumption, and cholesterol levels (26). In addition, patients with COVID-19 in Africa, older age, the presence of chronic disease, travel history, and the locations of Central Africa and West Africa were associated with increased mortality (27). Moreover, a higher prevalence of other communicable or non-communicable diseases (e.g., hypertension and diabetes) (28, 29), insufficient resuscitation beds and respirators, limited diagnosis capacity, and underreporting may have contributed to the higher CFR (21, 30).

Remarkably, our analysis found that at least eight SARS-CoV-2 variants were observed, with an obvious spatiotemporal profile during the four waves. In GHA, initial local transmission was dominated by the B.1.1 lineage, but the second wave was overwhelmingly driven by the Alpha variant. Subsequently, an unheralded variant under monitoring, B.1.1.318, dominated transmission from April to June 2021 before being displaced by Delta variants, which were introduced into the community in May 2021 (31). Remarkably, seven variants out of eight were initially identified outside West Africa, thus implicating that most SARS-CoV-2 variants in this region were introduced from different origins. An analysis has shown that the SARS-CoV-2 virus identified in Gambia is of European and Asian origin, and sequencing data matched patients' travel history (32). These analyses provided strong statistical support for a monophyletic origin of almost all of the Alpha sequences from NGA, with the most likely origin being the United States (33). Similarly, the Congolese SARS-CoV-2 sequences were divided into distinct clusters, indicating two separate introductions of the virus into the Republic of Congo (34). In this study, the first two waves were associated with a mix of SARS-CoV-2 lineages. The most severe wave was the second wave, included seven different variants. This is a potential cause for the severity of the second wave. The Eta variant originated in West Africa before spreading globally and represented a VOC in early 2021 (33), and this variant was distributed in 15 countries in West Africa. A previous study provided evidence of increased transmission for both Alpha and Eta variants, of which Eta appeared slightly more infectious than Alpha (35). These data reveal that the COVID-19 pandemic in West Africa was co-driven by both multiple introduced lineages and a single native lineage. In Southern Africa, the first wave was associated with a mix of SARS-CoV-2 lineages, while the second and third waves were driven by the Beta (B.1.351) and Delta (B.1.617.2) variants, respectively (23, 36). The two above-mentioned variants have immune evasion and higher transmissibility than previous variants (37). However, Africa's fourth pandemic wave was primarily driven by the Omicron variant. In this study, 21.7% of cases and the lowest number of deaths were observed in the shortest wave, the fourth wave, driven by Omicron. Under the same epidemiological conditions, a recent study found that the effective (instantaneous) reproduction number of Omicron is 3.19 (95% confidence interval [*CI*]: 2.82–3.61) times greater than that of Delta (38). The rate of infectivity of the Omicron variant is much higher than that of the Delta variant, and in a very short time, Omicron has displaced the Delta variant and has become the dominant variant across the globe (39). Similarly, Omicron infection is associated with significantly lower disease severity compared with the Delta variant, which has a striking immune escape ability (40, 41). Currently, Omicron variants BA.4 and BA.5 are emerging and are speculated to be more transmissible and resistant to immunity generated by previous variants such as Omicron BA.1 and most monoclonal antibodies (42). Moreover, asymptomatic transmission in the region was higher than symptomatic transmission (43), and asymptomatic individuals were the source of 69% (20–85%) of all infections (44). So, the control of COVID-19 in West African countries is still a great challenge. Furthermore, coverage of COVID-19 vaccination is low in African countries due to a lack of competence, infrastructure, logistics, and financial resources (45–47). We recommend that the region quickly increase the COVID-19 immunization coverage, enhance the genomic surveillance, improve testing, and strengthen entry surveillance to mitigate the spread of the infection (48).

Although the present analysis provides new insights to better understand COVID-19 epidemiology, some limitations are notable. First, data analyzed in the present study were obtained from public sources, which may not fully reflect the true COVID-19 epidemic situation on this continent due to incomplete data. Second, a relative univariate correlation analysis was conducted in our study; however, multivariate analysis can qualitatively determine variables in influence on the virus transmissibility, as well as better understanding of environmental factors driver on impact transmissibility for COVID-19 (49). Third, genome data were incomplete in the early stages of the pandemic. Sequence analysis of SARS-CoV-2 from different countries will provide evidence to monitor the COVID-19 spread pattern.

## Conclusion

In the present study, a comprehensive epidemiological retrospective analysis of COVID-19 was performed, with the purpose of better understanding the epidemiological features in West Africa from March 2020 to March 2022. There were four waves of COVID-19 in West Africa during this period, and the pandemic situation in these countries, which was co-driven by both multiple introduced variants and a native lineage, was highly varied. The continuous evolution of the COVID-19 pandemic in this continent is bound to give rise to the emergence of more new variants, and it is important to improve the genome surveillance capacity and prevent further spread of COVID-19.

## Data availability statement

The original contributions presented in the study are included in the article/Supplementary material, further inquiries can be directed to the corresponding authors.

## Author contributions

ZLiu and LG performed the data collection, processed the data, and drafted the manuscript. ZLiu, XD, and ZLi participated in the design of the study. QS and CZ critically reviewed the manuscript. LW, JN, and AT participated in the design of the study and also managed the project. All authors contributed to the article and approved the submitted version.

## Funding

This work was supported by the National Key R&D Program of China (Grant No. 2020YFE0205700), the Youth Science Foundation of the State Key Laboratory of Infectious Disease Prevention and Control, China CDC (Grant No. 2021SKLID503), the National Natural Science Foundation of China (Grant No. 82073624), and China-Sierra Leone Biosafety Laboratory Technical Cooperation Project (III Phase). The funders had no role in the study design, data collection and analysis, decision to publish, and preparation of the manuscript.

## Conflict of interest

The authors declare that the research was conducted in the absence of any commercial or financial relationships that could be construed as a potential conflict of interest.

## Publisher's note

All claims expressed in this article are solely those of the authors and do not necessarily represent those of their affiliated organizations, or those of the publisher, the editors and the reviewers. Any product that may be evaluated in this article, or claim that may be made by its manufacturer, is not guaranteed or endorsed by the publisher.

## References

[1] Lai CC Shih TP Ko WC Tang HJ Hsueh PR. Severe acute respiratory syndrome coronavirus 2 (SARS-CoV-2) and coronavirus disease-2019 (COVID-19): the epidemic and the challenges. Int J Antimicrob Agents. (2020) 55:105924. 10.1016/j.ijantimicag.2020.105924 32081636 PMC7127800

[2] Dufailu OA Afriyie-Asante A Gyan B Kwabena DA Yeboah H Ntiakoh F. COVID-19 in Africa: an ovarian victory? J Ovarian Res. (2021) 14:70. 10.1186/s13048-021-00820-1 34020688 PMC8138090

[3] Adepoju P. Nigeria responds to COVID-19; first case detected in sub-Saharan Africa. Nat Med. (2020) 26:444–8. 10.1038/d41591-020-00004-2 32161414

[4] Wale-Oshinowo BA Omobowale AO Adeyeye MM Lebura S. Least developed countries in Africa. In: Romaniuk S, Marton P, editors. The Palgrave Encyclopedia of Global Security Studies. Cham: Springer International Publishing (2020). p. 1–16. 10.1007/978-3-319-74336-3_346-1

[5] Martinez-Alvarez M Jarde A Usuf E Brotherton H Bittaye M Samateh AL. COVID-19 pandemic in west Africa. Lancet Glob Health. (2020) 8:e631–2. 10.1016/S2214-109X(20)30123-6 32246918 PMC7186549

[6] Tinto B Salinas S Dicko A Kagone TS Traore I de Rekeneire N. Spreading of SARS-CoV-2 in West Africa and assessment of risk factors. Epidemiol Infect. (2020) 148:e213. 10.1017/S0950268820002149 32921332 PMC7506176

[7] Alene KA Elagali A Barth DD Rumisha SF Amratia P Weiss DJ. Spatial codistribution of HIV, tuberculosis and malaria in Ethiopia. BMJ Glob Health. (2022) 7:e007599. 10.1136/bmjgh-2021-007599 35217531 PMC8867247

[8] Chanda-Kapata P Ntoumi F Kapata N Lungu P Mucheleng'anga LA Chakaya J. Tuberculosis, HIV/AIDS and malaria health services in sub-Saharan Africa—a situation analysis of the disruptions and impact of the COVID-19 pandemic. Int J Infect Dis. (2022). 10.1016/j.ijid.2022.03.033 [Epub ahead of print]. 35341998 PMC8949686

[9] Okereke M Ukor NA Ngaruiya LM Mwansa C Alhaj SM Ogunkola IO. COVID-19 misinformation and infodemic in rural Africa. Am J Trop Med Hyg. (2020) 104:453–6. 10.4269/ajtmh.20-1488 33382028 PMC7866344

[10] States ECoWA. Extreme poverty rises in West Africa due to COVID-19 pandemic. (2022). Available online at: https://www.wfp.org/news/extreme-poverty-rises-west-africa-due-covid-19-pandemic (accessed January 20, 2022).

[11] Bonnet E Bodson O Le Marcis F Faye A Sambieni NE Fournet F. The COVID-19 pandemic in francophone West Africa: from the first cases to responses in seven countries. BMC Public Health. (2021) 21:1490. 10.1186/s12889-021-11529-7 34340668 PMC8327893

[12] Giandhari J Pillay S Wilkinson E Tegally H Sinayskiy I Schuld M. Early transmission of SARS-CoV-2 in South Africa: an epidemiological and phylogenetic report. medRxiv. (2020) 103:234–41. 10.1016/j.ijid.2020.11.128 33189939 PMC7658561

[13] GICJNV. Covid-19 effects on Human Rights in West Africa. 2022. Available online at: https://www.gicj.org/positions-opinons/gicj-positions-and-opinions/2471-selling-livelihood-for-food-in-west-africa (accessed February 15, 2022).

[14] Salyer SJ Maeda J Sembuche S Kebede Y Tshangela A Moussif M. The first and second waves of the COVID-19 pandemic in Africa: a cross-sectional study. Lancet. (2021) 397:1265–75. 10.1016/S0140-6736(21)00632-2 33773118 PMC8046510

[15] Okonji EF Okonji OC Mukumbang FC Van Wyk B. Understanding varying COVID-19 mortality rates reported in Africa compared to Europe, Americas and Asia. Trop Med Int Health. (2021) 26:716–9. 10.1111/tmi.13575 33733568 PMC8251241

[16] Impouma B Williams GS Moussana F Mboussou F Farham B Wolfe CM. The first 8 months of COVID-19 pandemic in three West African countries: leveraging lessons learned from responses to the 2014-2016 Ebola virus disease outbreak. Epidemiol Infect. (2021) 149:e258. 10.1017/S0950268821002053 34493348 PMC8712942

[17] Udoakang AJ Djomkam Zune AL Tapela K Owoicho O Fagbohun IK Anyigba CA. Knowledge, attitude and perception of West Africans towards COVID-19: a survey to inform public health intervention. BMC Public Health. (2022) 22:445. 10.1186/s12889-022-12814-9 35248006 PMC8898084

[18] Tso FY Lidenge SJ Peña PB Clegg AA Ngowi JR Mwaiselage J. High prevalence of pre-existing serological cross-reactivity against severe acute respiratory syndrome coronavirus-2 (SARS-CoV-2) in sub-Saharan Africa. Int J Infect Dis. (2021) 102:577–83. 10.1016/j.ijid.2020.10.104 33176202 PMC7648883

[19] Milleliri JM Coulibaly D Nyobe B Rey JL Lamontagne F. Hocqueloux L, et al. SARS-CoV-2 infection in ivory coast: a serosurveillance survey among gold mine workers. Am J Trop Med Hyg. (2021) 104:1709–12. 10.4269/ajtmh.21-0081 33735104 PMC8103493

[20] Kangbai JB Sheku M Koroma B Macathy JM Kaitibi D Sahr F. African's COVID-19 third wave: a coupled behavior-disease system in a mutual feedback loop. Gazette Med Sci. (2021) 2:26–30. 10.46766/thegms.epidemiol.21101704

[21] Burki TK. Undetected COVID-19 cases in Africa. Lancet Respir Med. (2021) 9:e121. 10.1016/S2213-2600(21)00504-X 34774187 PMC8585486

[22] Renewal A. Omicron-fuelled COVID-19 surge in Africa plateaus. (2022). Available online at: https://www.un.org/africarenewal/magazine/february-2021/omicron-fuelled-covid-19-surge-africa-plateaus.

[23] Tegally H Wilkinson E Lessells RJ Giandhari J Pillay S Msomi N. Sixteen novel lineages of SARS-CoV-2 in South Africa. Nat Med. (2021) 27:440–6. 10.1038/s41591-021-01255-3 33531709

[24] Egbe KA Lerum NI Unah UV Ike AC Odoh CK Egbe FA. The biology of SARS-CoV-2 and epidemiological analysis of COVID-19 pandemic in Nigeria. J Infect Dev Ctries. (2022) 16:252–7. 10.3855/jidc.14467 35298418

[25] Liu Z Gao L Xue C Zhao C Liu T Tia A. Epidemiological trends of coronavirus disease 2019 in Sierra Leone from March 2020 to October 2021 [Original Research]. Front Public Health. (2022) 10:271. 10.3389/fpubh.2022.949425 35844842 PMC9276960

[26] Salom I Rodic A Milicevic O Zigic D Djordjevic M Djordjevic M. Effects of demographic and weather parameters on COVID-19 basic reproduction number [Original Research]. Front Ecol Evolut. (2021) 8:617841. 10.3389/fevo.2020.617841

[27] Mohammed M Muhammad S Mohammed FZ Mustapha S. Sha'aban A, Sani NY, et al. Risk factors associated with mortality among patients with novel coronavirus disease (COVID-19) in Africa. J Racial Ethn Health Disparities. (2021) 8:1267–72. 10.1007/s40615-020-00888-3 33051749 PMC7553376

[28] Bank AD. Health in Africa over the next 50 years. (2013).

[29] Mudie K Jin MM Tan Kendall L Addo J Dos-Santos-Silva I Quint J. Non-communicable diseases in sub-Saharan Africa: a scoping review of large cohort studies. J Glob Health. (2019) 9:020409. 10.7189/jogh.09.020409 31448113 PMC6684871

[30] Teymouri M Mollazadeh S Mortazavi H Naderi Ghale-Noie Z Keyvani V Aghababaei F. Recent advances and challenges of RT-PCR tests for the diagnosis of COVID-19. Pathol Res Pract. (2021) 221:153443. 10.1016/j.prp.2021.153443 33930607 PMC8045416

[31] Morang'a CM Ngoi JM Gyamfi J Amuzu DSY Nuertey BD Soglo PM. Genetic diversity of SARS-CoV-2 infections in Ghana from 2020–2021. Nat Commun. (2022) 13:2494. 10.1038/s41467-022-30219-5 35523782 PMC9076825

[32] Kanteh A Manneh J Jabang S Kujabi MA Sanyang B Oboh MA. Origin of imported SARS-CoV-2 strains in The Gambia identified from whole genome sequences. PLoS ONE. (2021) 16:e0241942. 10.1371/journal.pone.0241942 34464385 PMC8407536

[33] Ozer EA Simons LM Adewumi OM Fowotade AA Omoruyi EC Adeniji JA. Multiple expansions of globally uncommon SARS-CoV-2 lineages in Nigeria. Nat Commun. (2022) 13:688. 10.1038/s41467-022-28317-5 35115515 PMC8813984

[34] Ntoumi F Mfoutou Mapanguy CC Tomazatos A Pallerla SR Linh LTK Casadei N. Genomic surveillance of SARS-CoV-2 in the Republic of Congo. Int J Infect Dis. (2021) 105:735–8. 10.1016/j.ijid.2021.03.036 33737129 PMC7959680

[35] Zhao S Musa SS Chong MK Ran J Javanbakht M Han L. The co-circulating transmission dynamics of SARS-CoV-2 Alpha and Eta variants in Nigeria: a retrospective modeling study of COVID-19. J Glob Health. (2021) 11:05028. 10.7189/jogh.11.05028 35136591 PMC8801210

[36] Tegally H Wilkinson E Giovanetti M Iranzadeh A Fonseca V Giandhari J. Detection of a SARS-CoV-2 variant of concern in South Africa. Nature. (2021) 592:438–43. 10.1038/s41586-021-03402-9 33690265

[37] Mlcochova P Kemp SA Dhar MS Papa G Meng B Ferreira IATM. et al. SARS-CoV-2 B16172 Delta variant replication and immune evasion. Nature. (2021) 599:114–9. 34488225 10.1038/s41586-021-03944-y PMC8566220

[38] Ito K Piantham C Nishiura H. Relative instantaneous reproduction number of Omicron SARS-CoV-2 variant with respect to the delta variant in Denmark. J Med Virol. (2022) 94:2265–8. 10.1002/jmv.27560 34967453 PMC9015237

[39] Mohapatra RK Tiwari R Sarangi AK Sharma SK Khandia R Saikumar G. Twin combination of Omicron and Delta variants triggering a tsunami wave of ever high surges in COVID-19 cases: a challenging global threat with a special focus on the Indian subcontinent. J Med Virol. (2022) 94:1761–5. 10.1002/jmv.27585 35014038 PMC9015634

[40] Butt AA Dargham SR Tang P Chemaitelly H Hasan MR Coyle PV. COVID-19 disease severity in persons infected with the Omicron variant compared with the Delta variant in Qatar. J Glob Health. (2022) 12:05032. 10.7189/jogh.12.05032 35788085 PMC9253930

[41] Ma C Chen X Mei F Xiong Q Liu Q Dong L. Drastic decline in sera neutralization against SARS-CoV-2 Omicron variant in Wuhan COVID-19 convalescents. Emerg Microbes Infect. (2022) 11:567–72. 10.1080/22221751.2022.2031311 35060426 PMC8856021

[42] Shrestha LB Foster C Rawlinson W Tedla N Bull RA. Evolution of the SARS-CoV-2 omicron variants BA1 to BA5: Implications for immune escape and transmission. Rev Med Virol. (2022) 20:e2381. 10.1002/rmv.2381 35856385 PMC9349777

[43] Taboe HB Salako KV Tison JM Ngonghala CN Glèlè Kakaï R. Predicting COVID-19 spread in the face of control measures in West Africa. Math Biosci. (2020) 328:108431. 10.1016/j.mbs.2020.108431 32738248 PMC7388784

[44] Emery JC Russell TW Liu Y Hellewell J Pearson CA Knight GM. The contribution of asymptomatic SARS-CoV-2 infections to transmission on the Diamond Princess cruise ship. Elife. (2020) 9:e58699. 10.7554/eLife.58699 32831176 PMC7527238

[45] Ariyo OE Oladipo EK Osasona OG Obe O Olomojobi F. COVID-19 vaccines and vaccination: how prepared is Africa? Pan Afr Med J. (2021) 39:107. 10.11604/pamj.2021.39.107.27912 34512843 PMC8396376

[46] Jerving S. The long road ahead for COVID-19 vaccination in Africa. Lancet. (2021) 398:827–8. 10.1016/S0140-6736(21)01967-X 34481563 PMC8412836

[47] Lawal L Aminu Bello M Murwira T Avoka C. Yusuf Ma'aruf S, Harrison Omonhinmin I, et al. Low coverage of COVID-19 vaccines in Africa: current evidence and the way forward. Hum Vaccin Immunother. (2022) 18:2034457. 10.1080/21645515.2022.2034457 35240908 PMC9009957

[48] Lokossou VK Umeokonkwo CD Okolo S Nguku PM Ogbureke N Sombie I. COVID-19 pandemic waves: how prepared is West Africa for managing a high COVID-19 caseload? Urgent actions needed. Pan Afr Med J. (2021) 40:249. 10.11604/pamj.2021.40.249.31107 35233269 PMC8831218

[49] Djordjevic M Salom I Markovic S Rodic A Milicevic O Djordjevic M. Inferring the main drivers of SARS-CoV-2 global transmissibility by feature selection methods. Geohealth. (2021) 5:e2021GH000432. 10.1029/2021GH000432 34568708 PMC8448988

